# Early Palliative Care—Health services research and implementation of sustainable changes: the study protocol of the EVI project

**DOI:** 10.1186/s12885-015-1453-0

**Published:** 2015-05-29

**Authors:** Cornelia Meffert, Jan Gaertner, Katharina Seibel, Karin Jors, Hubert Bardenheuer, Dieter Buchheidt, Regine Mayer-Steinacker, Marén Viehrig, Christina Paul, Stephanie Stock, Carola Xander, Gerhild Becker

**Affiliations:** 1 Department of Palliative Care, Comprehensive Cancer Center, University Medical Center Freiburg, Freiburg, Germany; 2 Palliative Care Center of Excellence for Baden-Wuerttemberg, Baden-Wuerttemberg, Germany; 3 Department of Anesthesiology, Comprehensive Cancer Center, University Medical Center Heidelberg, Heidelberg, Germany; 4 Department of Hematology and Oncology, Comprehensive Cancer Center, Mannheim University Hospital, University of Heidelberg, Heidelberg, Germany; 5 Department of Hematology and Oncology, Comprehensive Cancer Center, University Medical Center Ulm, Ulm, Germany; 6 Department of Radiation Oncology, Comprehensive Cancer Center, University Medical Center Tuebingen, Tuebingen, Germany; 7 Paul-Lechler Hospital Tuebingen, Tuebingen, Germany; 8 Institute of Health Economics and Clinical Epidemiology, University Clinic of Cologne (AöR), Cologne, Germany; 9 Department of Palliative Care, Palliative Care Research Group, University Medical Center Freiburg, Robert-Koch-Str. 3, 79106 Freiburg, Germany

**Keywords:** Cancer, Terminally ill patients, Early palliative care

## Abstract

**Background:**

International medical organizations such as the American Society of Medical Oncology recommend early palliative care as the “gold standard” for palliative care in patients with advanced cancer. Nevertheless, even in Comprehensive Cancer Centers, early palliative care is not yet routine practice. The main goal of the EVI project is to evaluate whether early palliative care can be implemented—in the sense of “putting evidence into practice”—into the everyday clinical practice of Comprehensive Cancer Centers. In addition, we are interested in (1) describing the type of support that patients would like from palliative care, (2) gaining information about the effect of palliative care on patients’ quality of life, and (3) understanding the economic burden of palliative care on patients and their families.

**Methods/design:**

The EVI project is a multi-center, prospective cohort study with a sequential control group design. The study is a project of the Palliative Care Center of Excellence (KOMPACT) in Baden-Württemberg, Germany, which was recently established to combine the expertise of five academic, specialist palliative care departments. The study is divided into two phases: preliminary phase (months 1–9) and main study phase (months 10–18). In each of all five participating academic Comprehensive Cancer Centers, an experienced palliative care physician will be hired for 18 months. During the preliminary phase, the physician will be allowed time to establish the necessary structures for early palliative care within the Comprehensive Cancer Center. In the main study phase, patients with metastatic cancer will be offered a consultation with the palliative care physician within eight weeks of diagnosis. After the initial consultation, follow-up consultations will be offered as needed. The study is built upon a convergent parallel design. In the quantitative arm, patients will be surveyed in both the preliminary and main study phase at three points in time (baseline, 12 weeks, 24 weeks). Standardized questionnaires will be used to measure patients’ quality of life, symptom burden and mood. Using interviews with palliative care physicians, oncologists, department heads, patients and their caregivers, the qualitative arm will explore (1) what factors encourage and hinder the early integration of palliative care into standard oncology care, (2) what support patients and their caregivers would like from palliative care, and (3) what effect palliative care has on the economic disease burden of patients and their families.

**Discussion:**

The study proposed is meant to serve as a catalyzer. Local palliative care teams should be put in position to routinely cooperate with the primary treating department at their respective cancer center. The long-term goal of this project is to create sustainable improvements in the care of patients with incurable cancer.

**Trial registration:**

DRKS00006162; date of registration: 19/05/2014

## Background

The primary goal of palliative care (PC) is to ensure quality of life (QoL) for patients with incurable, life-limiting diseases and their caregivers. This is true for all patients, regardless of their disease (e.g. amyotrophic lateral sclerosis, multiple sclerosis, heart failure, chronic obstructive pulmonary disorder, etc.) However, patients with incurable cancer remain one of the largest patient groups with a need for palliative care [1]. Due to the continuing advances in cancer treatment, cancer is likely to increasingly become a chronic disease [2].

In the past and even today, PC has often been falsely equated with end-of-life care [3]. For this reason, treatment goals in standard oncology care have often focused solely on prolonging life rather than improving QoL early in the course of the disease [4]. Even in early stages of the disease, patients with incurable cancer often suffer from multiple physically burdensome symptoms (e.g. pain) [5] and psychosocial and spiritual anxiety. By focusing on life-prolonging treatment options, the suffering that patients and their families experience from these symptoms often goes unnoticed and is not adequately relieved [3]. The outdated dictum “first prolong life, then improve quality of life” should be replaced by a holistic, integrative model [6]. This approach is referred to as early palliative care (EPC). According to this model, specialist palliative care expertise focused on the needs of the patient should be offered alongside standard oncology care [7].

For patients with non-small cell lung cancer (NSCLC), Temel et al. [8] showed that EPC improved patients’ QoL, reduced the incidence of depression, and decreased the number of aggressive (and generally futile) therapies at the end of life. In addition, EPC increased patients’ survival time [8]. In a randomized study with patients suffering from different cancer entities, Zimmermann et al. [9] found that patients who received EPC not only had a better QoL but also a higher level of satisfaction with their treatment compared to those in the control group, who were not offered EPC.

Meanwhile, international medical organizations such as the American Society of Medical Oncology (ASCO) have recommended EPC as the “gold standard” for palliative care in patients with advanced cancer [3]. However, integrating palliative care into standard oncology care early in the course of the disease requires a reorientation and restructuring of current medical practice. For this reason, EPC has not yet become a routine part of care even in Comprehensive Cancer Centers (CCCs) [3].

In spite of their clear recommendation for EPC, the National Cancer Institute and the ASCO also point out that there are considerable deficits in the currently available publications on EPC [3]. These institutions criticize that studies have focused on limited patient populations (e.g. patients with NSCLC) rather than the entire spectrum of cancer patients who are actually treated in CCCs. Furthermore, recent studies have been criticized because they have been conducted under ideal circumstances, which do not correspond to the real-life situation. This makes it impossible to assess the feasibility and effectiveness of EPC in the actual clinical setting [10]. Until now, there has been a lack of research investigating the implementation of EPC into everyday clinical practice, using the already available resources and structures [11]. In light of this, the study proposed here aims to implement EPC into clinical practice at all five CCCs throughout the federal state of Baden-Württemberg, Germany. The study is a project of the Palliative Care Center of Excellence (KOMPACT) in Baden-Württemberg, Germany. It is funded by the Robert Bosch Foundation who has formally peer reviewed our study protocol before assigning the grant. Patients with advanced metastatic cancer that is unresponsive to curative treatments will routinely be offered palliative care as soon as possible after diagnosis, according to the most up-to-date international treatment standards.

The main goal of the EVI project is to evaluate whether EPC can be implemented into the everyday clinical practice of CCCs, and if so, what conditions are necessary for it to succeed. In addition, we are interested in (1) describing the type of support that patients would like from palliative care, (2) gaining information about the effect of palliative care on patients’ QoL and healthcare costs, and (3) determining which factors could potentially encourage or hinder the implementation of EPC, respectively.

## Methods/design

### The design of the study

In the field of PC, conducting clinical studies is associated with considerable challenges. Reasons for this include (1) a lack of established scientific standards for PC research questions and (2) the complex medical and personal situation of PC patients, which places unique demands on the design of studies with this patient population [12]. Only recently was a guidance statement for end-of-life care research developed in the context of the “Methods of Researching End of Life Care” (MOREcare) project, which expanded on the Medical Research Council guidance on the development and evaluation of complex circumstances [13]. The design of the study proposed here closely follows the guidelines established by the MOREcare project. Another important underlying premise of our project is its real-world design, which involves the routine implementation of EPC in the clinical setting and helps avoid artificial study conditions.

The study is divided into two phases: preliminary phase (months 1–9) and main study phase (months 10–18). In each of the five participating CCCs, an experienced palliative care physician will be hired for 18 months with a 50 % position. During the preliminary phase of the study, the physician will be allowed time to establish the necessary structures for EPC within the clinic. In addition, patients will also be recruited during this phase to establish a reference group for comparison between the status quo and those in the main study phase who receive EPC.

In the main study phase, patients with metastatic cancer will routinely be offered a consultation with the PC physician within eight weeks of diagnosis. This initial consultation has multiple objectives. First, this meeting serves to provide information regarding the value and accessibility of specialist palliative care. The PC physician will explain to patients that interdisciplinary cancer treatment ensures that all meaningful treatment options will continue to be available (“fight against cancer”) but that high priority will be placed on QoL also. For QoL needs, specialist palliative care services will be available to patients alongside treatment from the primary cancer specialist. It is important to make clear to patients that EPC is supplementary to standard oncology care and aims at supporting the treating cancer specialist. The cancer specialist will remain the primary contact person regarding patients’ treatment options.

The second main objective of the initial consultation is to create a certain familiarity with palliative care, which will help reduce barriers to accessing these services in the future.

In the initial consultation, a detailed assessment of patients’ physical symptoms (e.g. pain), psychosocial issues, spiritual burden and information needs will be conducted using a combination of both structured and unstructured methods. If an intervention appears necessary (e.g. adjusting medications), suggestions will be provided to the primary treating physician and, when possible, will be carried out directly in the outpatient clinic of the CCC. In these cases, a follow-up consultation will be arranged between the patient and PC physician. Otherwise, patients and treating physicians have the possibility to contact the PC physician as necessary. Follow-up consultations are therefore not obligatory. Rather, additional meetings will take place as needed, according to the requests of patients, family members/caregivers or the treating physician.

In order to ensure that the EPC services in all five CCCs are comparable, the palliative care physicians will receive a special structured training during the preliminary phase of the study. In an earlier project, a leading researcher from our working group established which factors are important for the early implementation of palliative care [7]. Based on this, a method was developed to improve physicians understanding of the initial PC consultation and cooperation between various medical specialties [14]. This method will serve as the basis of our training for PC physicians in this study.

Before the main study phase begins, cancer specialists at each center will be comprehensively informed about the possibilities of EPC so that they are able to prepare patients for the PC consultation. We will spread information about EPC by distributing informational brochures, sending a regular newsletter to physicians and nurses, and by holding presentations to the tumor boards of each participating center.

To evaluate the feasibility of the routine implementation of EPC in CCCs, the study is built upon a convergent parallel design [15]. The quantitative arm is conceived as a multi-center, prospective cohort study with a sequential control group design. Both in the preliminary and main study phase, patients will be surveyed at three points in time (baseline, 12 weeks, 24 weeks). Standardized questionnaires will be used to measure patients’ QoL, symptom burden and mood. The economic effects of EPC will be measured using an expense log or cost diary.

In light of the objective to test the feasibility of EPC, the qualitative arm of the study consist of four interview studies with palliative care physicians, oncologists/department heads, patients and their families/caregivers. These interviews will explore: (1) which factors encourage and hinder the early integration of palliative care into standard oncology care and (2) what support patients and their families would like from palliative care and to what extent these needs can be met through the EPC approach used in this study.

After conclusion of the study, the results of the quantitative and qualitative arms will be synthesized and evaluated with regard to the feasibility of EPC in CCCs.

### Quantitative arm

#### Participants

The EPC services provided in this study are aimed at patients with advanced metastatic cancer that is unresponsive to curative treatments (ICD 10 C 1–80 plus ICD 10 C 78–79). In all participating CCCs, patients will be identified in the tumor boards of each center. As soon as the diagnostic process has been concluded and treatment has started (i.e. within the first eight weeks after diagnosis), patients will be referred to the PC physician. Table 1 shows the inclusion and exclusion criteria for participation.

**Table 1:** Inclusion and exclusion criteria for participation in the quantitative study arm

Inclusion criteria:	
1.	Initial diagnosis of a metastatic, incurable cancer (ICD 10 C 1–80 plus ICD 10 C 78–79) occurred within the last eight weeks, particularly:
	- non-small cell lung cancer (NSCLC) without epidermal growth factor (EFGR) mutations: met. NSCLC Stage IV – ICD C34.[01239] + metastasis code or C34.8 (multiple subdomains) + possibly M8012/3 (large cell carcinoma)
	- met. esophageal carcinoma Stage IV – ICD C15.[123459] + metastasis code or C15.8 (multiple subdomains) + possibly M8070/3 (squamous cell carcinoma)
	- met. stomach carcinoma Stage IV - ICD C16.[01234569] + metastasis code or C16.8 (multiple subdomains) + possibly M8145/3 (adenocarcinoma, diffuse)
	- non-endocrine pancreas carcinoma Stage IV - ICD C25.[012379] + metastasis code or C25.8 (multiple subdomains) + possibly M8971/3 (pancreas blastoma)
	- center-specific tumor entities
2.	Age ≥ 18 years
3.	Ability to understand written and verbal questions in German
4.	Willingness to participate in the study
5.	Informed consent
Exclusion criteria:	
1.	Other hemato-oncological disease (e.g. leukemia)
2.	Dementia
3.	Psychosis/delirium
4.	Major depression

### Outcome assessment

We will consider EPC to be feasible if 75 % of all eligible patients (i.e. adult patients diagnosed with an incurable, metastatic cancer [ICD 10 C 1–80 plus ICD 10 C 78–79]) are referred to a PC physician at their center at least once within eight weeks of the initial diagnosis.

Patients’ QoL and symptom burden will be assessed at the initial PC consultation using the “Palliative Outcome Scale” (POS) [16], the “European Organization for Research and Treatment of Cancer (EORTC) QLQ-C30” [17], and the “Hospital Anxiety and Depression Scale” (HADS) [18]. Both in the preliminary and the main study phase, a follow-up assessment will be conducted at 12 and 24 weeks using these three instruments. Family/caregivers of the patient will be asked to assess the patients’ situation by filling out the “Quality of Dying and Death” questionnaire [19].

The effects of EPC on healthcare costs will be measured in part by the number of follow-up visits a patient requires after the initial PC consultation, as recorded by the PC physician. In addition, patients will be asked to keep track of any additional costs in an expense log/cost diary. The expense log/cost diary is a bottom-up approach to understand direct and indirect costs of care from a patient perspective and a societal perspective. It will allow us to follow which kind of health-related services/treatments are used including services not reimbursed by the Statutory Health Insurance, how much productivity of patients and caregivers is lost and how much patients and their families spend on out-of-pocket payments. Accordingly, patients and their caregivers will be instructed to quantify all health-related costs and the reason for the costs in a provided expense log/cost diary. The expense log/cost diary will be developed based on a literature review and interviews with patients and their families. It will be pretested extensively before routine use.

### Quantitative data collection in the preliminary and main study phase

In each participating CCC, a study coordinator will be appointed. As soon as a patient is identified in the tumor board as a potential participant, the study coordinator will contact the patient, provide the patient with information and ask for the patient’s consent to participate in the study. If the patient is willing to participate, the study coordinator will explain the expense log/cost diary and hand it out to the patient. He will also ask the patient to fill out the T0-questionnaires. The T0-questionnaires (i.e. POS, EORTC, QLQ-C30 and HADS) will be added to the patient file and will therefore be accessible to the treating physician. After 12 weeks, the patient will be contacted again by the study coordinator and will be asked to complete the T1-questionnaires. At the same time, the study coordinator will remind the patient to regularly fill out the expense log. A second follow-up assessment will occur at 24 weeks, and the patient will be asked to complete the T2-questionnaires. Also, the patient will be asked to return the completed expense log.

The patient burden in the last days of life will be assessed externally by a family member or most relevant caregiver of the patient. After the patient’s death, the family member/caregiver will be contacted and asked to complete the “Quality of Dying and Death” questionnaire via telephone. If the patient has not died by the end of the study phase, this questionnaire will not be filled out.

**Fig. 1:**
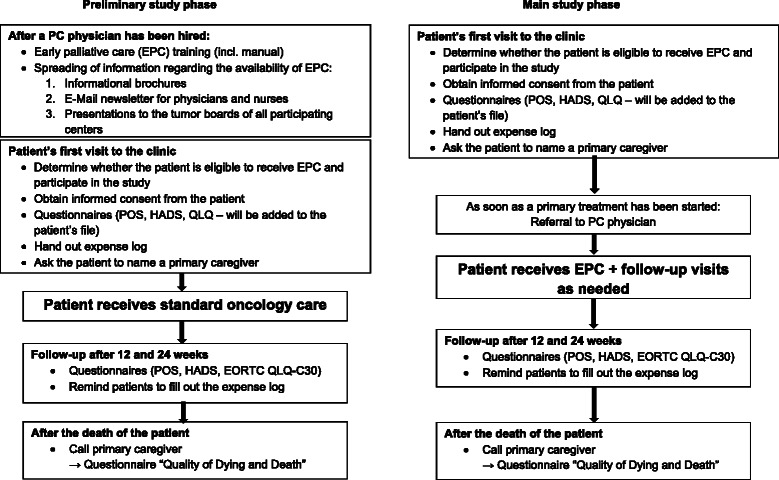
Course of events for both quantitative arms

**Fig. 2:**
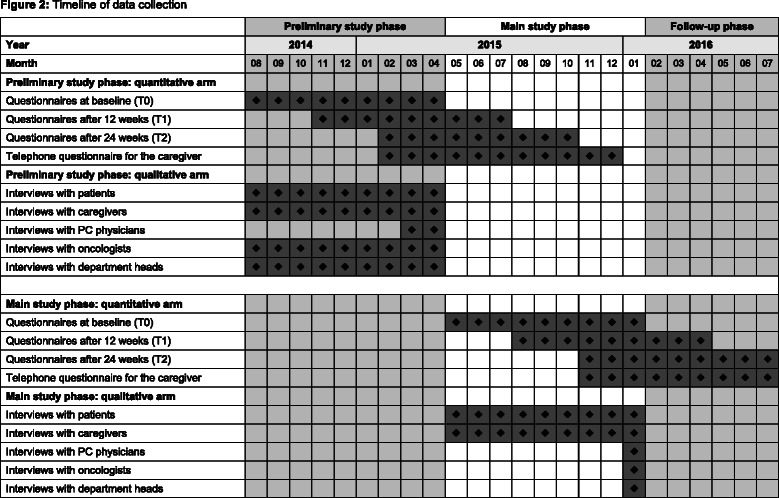
Timeline of data collection

The following figures show the course of events for both quantitative arms and the timeline for data collection for the quantitative and qualitative arms (Figs. 1 and 2).

### Quantitative data analysis

To judge the feasibility of the routine integration of EPC into clinical practice at CCCs, we will measure how many eligible patients were referred to the center’s PC physician within the first eight weeks after diagnosis. For this, we will rely on the internal records of the participating centers.

With an expected response rate of ca. 50 %, we aim for a sample size of 1000 patients (200 per participating CCC) in the preliminary study phase and an additional 1000 patients (200 per participating CCC) in the main study phase. Differences between the control group (patients in the preliminary study phase) and the intervention group (patients in the main study phase) will be analyzed with respect to symptom burden, QoL, and mood. The longitudinal data (i.e. at 12 and 24 weeks) will be analyzed in regard to changes between the baseline measurement and follow-up measurements. Effect sizes between 0.20 and 0.49 will be judged as small, between 0.50 and 0.79 as medium, and above 0.80 as large [20]. We will use descriptive data analysis along with t-tests, chi-square tests, Mann–Whitney-U tests, and multivariate linear and binary logistical regression models. All statistical tests will be two-sided, and p-value ≤ 0.05 will be considered significant. IMB SPSS (Version 21.0) will be used to conduct data analysis.

### The qualitative arm

#### Participants and sampling

The qualitative arm of the EVI project consists of four interview studies (Table 2). Interview partners for each study will be recruited equally from each participating CCC.

**Table 2:** Planned qualitative interviews at each participating CCC

	Preliminary phase:	Main phase:
	Number of interviews per center	Number of interviews per center
Palliative care physicians	1	1
Oncologists	5	5
Department heads	5	5
Patients	5	5
Family/caregivers	5	5
Total	21	21

##### Interview study with PC physicians

The five PC physicians hired for the project will be interviewed both in the preliminary and main study phase regarding the feasibility of integrating EPC into standard oncology care. In addition, the PC physicians will be asked what factors support and hinder the process of establishing EPC in their respective center.

##### Interview study with oncologists and department heads

During the preliminary and the main study phase, we will conduct expert interviews with 25 oncologists and 25 department heads. The focus of these interviews will be the feasibility of the routine integration of EPC into standard oncology care. The sample of oncologists and department heads will be achieved by using maximum variation sampling.

##### Interview study with patients and family/caregivers

During the preliminary and the main study phase, 25 patients and 25 family members/caregivers will be interviewed regarding what kind of support they would like from palliative care in addition to standard oncology care and how they perceive the provided EPC. The patient and caregiver samples will also be achieved by using maximum variation sampling. All patients and caregivers will be recruited from the quantitative arm of the study.

In total, we aim at 210 interviews for the qualitative arm of the project.

### Outcome assessment

To adequately assess the feasibility of integrating EPC into standard oncology care, it would be insufficient to only measure whether 75 % of all eligible patients are referred to a PC physician. Rather, it is essential to evaluate what circumstances make it possible (or not) to implement EPC. For this reason, the qualitative arm of this study will assess the strengths and weaknesses of this model of EPC along with potential areas of improvement from the perspective of all stakeholders (PC physicians, oncologists/department heads, patients and caregivers).

### Qualitative data collection in the preliminary and main study phase

The responsible research assistants from the Department of Palliative Care in Freiburg will work in close cooperation with the PC physician at each CCC to coordinate logistics and dates for the interviews in each of the four interview studies. The interviews will be conducted by interviewers who have received a training in qualitative interview methods.

### Qualitative data analysis

The guided interviews will be analyzed after transcription using the principles of qualitative content analysis. A team of researchers will inductively develop a category system in order to create a subject matrix for each of the four interview studies [21]. Content analysis will be aided by the use of the software program MAXQDA.

### Ethics

The study has been designed according to the Declaration of Helsinki [22]. The study protocol was approved by the Ethics Committee of the University of Freiburg, Germany (vote: 193/14, date: 25 April 2014) and the responsible data protection officer. In addition, ethical approval was obtained from the Ethics Committee of the University of Tuebingen, the Ethics Committee of the Mannheim University Hospital, the Ethics Committee of the University Medical Center Heidelberg, and the Ethics Committee of the University of Ulm. Consistent with good clinical practice, patients will be informed about participation in the study, its implications and written consent will be obtained.

## Discussion

The primary objectives of the implementation of EPC in our study are (1) to identify patients with unmet palliative care needs, (2) to advise patients regarding the possibilities of specialized PC in their particular situation, (3) to reduce inhibitions to make use of palliative care as a normal part of the center’s treatment options, (4) to create a “double awareness” for the will to survive (“fight against cancer”) and inevitable mortality, and (5) to prevent that PC is misunderstood as only end-of-life care.

Concerns have repeatedly been raised that cancer treatment will become disintegrated if treatment is too interdisciplinary [23]. Therefore, EPC must occur in close cooperation and communication with all other responsible departments (e.g. the oncology department). Above all, it is important that the patient maintains a feeling of security and does not become confused by conflicting messages regarding prognosis and treatment goals. Patients and their family/caregivers must always be aware who is primarily responsible for their care (typically this will be a physician from the oncology, radiation therapy or senology department) [11]. The model of EPC implemented in this study takes this premise very seriously. Based on clinical and administrational experiences from our EPC pilot study [7], we are convinced that EPC can only be successfully integrated into standard oncology care when there is close cooperation between PC and cancer specialists [24]. This goal also serves to establish clear areas of responsibility for everyone involved. Accordingly, the PC physician advises the patient, his family and the primary treating physician but refrains from becoming involved in decision-making regarding disease-modifying therapies. The most basic principle of this approach is to avoid conflicting messages and establish a trustful relationship between the patient and the primary physician.

The main goal of the study proposed here is to test the feasibility of implementing a routine EPC program into the standard oncology care of CCCs. In addition, we are interested in 1) describing the type of support that patients would like from palliative care, (2) gaining information about the effect of palliative care on patients’ QoL and healthcare costs, and (3) determining which factors could potentially ease or hinder the implementation of EPC, respectively. The long-term goal of this project is to create sustainable improvements in the care of patients with incurable, life-limiting cancer.

From the perspective of health services research, so-called “real-world” studies are urgently needed to gather accurate information about the effects of complex interventions (e.g. implementation of EPC) outside of artificial study conditions [25]. To our knowledge, no (inter‐)national study until now has investigated the feasibility – in the sense of “putting evidence into practice” – of the routine integration of EPC into everyday clinical practice at CCCs.

Synergistic, relevant effects from this project have the potential to lead to sustainable improvements in the care of patients with life-limiting illnesses. Thus, this project should serve as a catalyzer. Local palliative care teams should be put in position to routinely cooperate with the primary treating department at their respective cancer center. In addition, this project aims at publicly raising awareness for the need of supportive palliative care, aimed at accompanying patients throughout their care and reducing unnecessary suffering. Further, valuable information regarding the design of future studies and improvements in patient care can be gained from this study.

## Abbreviations

PC: Palliative Care EPC Early Palliative Care
NSCLC: Non-Small Cell Lung Cancer
QoL: Quality of life
ASCO: American Society of Medical Oncology
CCC: Comprehensive Cancer Center
POS: Palliative Outcome Scale
EORTC: European Organization for Research and Treatment of Cancer
HADS: Hospital Anxiety and Depression Scale
